# Design of a W-Band Low-Voltage TWT Utilizing a Spoof Surface Plasmon Polariton Slow-Wave Structure and Dual-Sheet Beam

**DOI:** 10.3390/s25185641

**Published:** 2025-09-10

**Authors:** Gangxiong Wu, Ruirui Jiang, Jin Shi

**Affiliations:** 1 School of Information Science and Technology, Nantong University, Nantong 226019, China; wugangxiong@163.com (G.W.); jinshi0601@hotmail.com (J.S.); 2 School of Microelectronics and Integrated Circuits, Nantong University, Nantong 226019, China

**Keywords:** W-band, traveling-wave tube, low-voltage, millimeter-wave power amplifier, beam-wave interaction

## Abstract

This paper presents a W-band low-voltage traveling-wave tube (TWT) incorporating a spoof surface plasmon polariton (SSPP) slow-wave structure (SWS) and a dual-sheet beam. The SSPP-based SWS adopts a periodic double-F-groove configuration, which provides strong field localization, increases the interaction impedance, and reduces the phase velocity, thereby enabling a low synchronization voltage. Owing to its symmetric open geometry, the SWS naturally forms a dual-sheet beam tunnel, which enhances the effective beam current without increasing the aperture size. Eigenmode calculations indicate that, within the 92–97 GHz band, the normalized phase velocity is between 0.198 and 0.208, and the interaction impedance exceeds 2.65 Ω. Moreover, an energy-coupling structure was developed to ensure efficient signal transmission. Three-dimensional particle-in-cell (PIC) simulations predict a peak output power of 366.1 W and an electronic efficiency of 6.15% at 95.5 GHz for a 2 × 250 mA dual-sheet beam at 11.9 kV, with stable amplification and without self-oscillation observed. The proposed low-voltage, high-efficiency W-band TWT offers a manufacturable and easily integrable solution for next-generation millimeter-wave systems, supporting high-capacity wireless backhaul, airborne communication, radar imaging, and sensing platforms where compactness and reduced power-supply demands are critical.

## 1. Introduction

With the explosive growth of millimeter-wave (MMW) wireless communication technologies and the surging demand for ultra-high-speed data transmission, increasingly stringent requirements are being imposed on the power capacity, bandwidth, and efficiency of power source devices [1,2]. Traveling-wave tubes (TWTs) have emerged as indispensable for MMW communication applications due to their superior power-handling capabilities, which significantly exceed those of solid-state amplifiers, as well as their inherent ability to simultaneously achieve high output power, gain, and wide bandwidth without significant trade-offs [3,4,5]. Owing to this advantage, TWTs are regarded as one of the most promising candidates for high-capacity wireless communication backhaul, airborne and vehicular high-speed data links, and next-generation MMW systems.

The W-band, particularly around 94 GHz, is recognized as a frequency window due to its low atmospheric attenuation and excellent anti-interference properties, rendering it highly suitable for long-distance, high-data-rate transmission [6,7]. However, current W-band TWTs still face several critical challenges, including limited output power, high operating voltage, bulky structures, and difficulties in microfabrication and assembly. Most notably, achieving sufficient beam–wave interaction and high output power, existing designs often require operating voltages above 20 kV [8,9,10,11,12,13,14,15,16,17,18]. Such high voltages not only complicate the supporting power supply and insulation design but also increase the system’s size, cost, and risk of failure, thereby hindering deployment in lightweight and mobile platforms. Furthermore, the conventional slow-wave structures (SWSs), such as folded waveguides (FWGs), sine waveguides (SWGs), or staggered grating waveguides (SGWs), typically exhibit low interaction impedances in the range of 1.5 to 3.0 Ω [11,12,15,19,20]. These structures rely on a single electron beam, resulting in reduced interaction efficiency, particularly under low-voltage operation, and insufficient output power. Additionally, the sub-millimeter periodicity required for W-band operation imposes significant fabrication challenges. Achieving the necessary dimensional accuracy during processing and assembly is critical to avoiding mode mismatches or beam truncation, either of which can severely degrade device performance.

To overcome these limitations, recent studies have shown that MTM-based concepts can be effectively applied in vacuum electron devices [19,20,21,22,23,24], such as dumbbell-shaped slot resonator slow-wave structures with metamaterial properties for W-band TWT amplifiers [19], double-negative metamaterial-loaded helices for dispersion tailoring and miniaturization [22], and comb-type SWSs for efficient low-voltage operation [23]. In addition, spiral metamaterials have been employed in forward-wave oscillators to realize high power and efficiency [24]. These advances confirm the potential of MTM-inspired designs to enhance stability, reduce operating voltage, and improve output power in next-generation TWTs. Another promising approach is based on spoof surface plasmon polariton (SSPP) configurations [25,26]. SSPP-based structures support subwavelength electromagnetic field confinement and exhibit favorable dispersion properties, making them well-suited to MMW applications. Compared with conventional designs, SSPP-based SWSs are expected to offer significantly higher interaction impedances and lower normalized phase velocities, while maintaining a simple all-metallic geometry that is conducive to scalable and cost-effective manufacturing.

In this work, we propose a novel W-band low-voltage TWT that integrates an SWS based on SSPPs with a dual-sheet beam configuration. The SSPP structure is specifically optimized to enhance interaction impedance and ensure phase synchronization with low-voltage electron beams, thereby reducing the required operating voltage without compromising performance. The dual-sheet beam configuration increases the effective beam current without enlarging the tunnel aperture, contributing to enhanced output power under low-voltage operation. Additionally, the all-metallic planar geometry facilitates fabrication via precision micromilling, MEMS, or LIGA processes, while the compact total length (<50 mm) supports integration into size- and weight-constrained platforms. This approach addresses the fundamental limitations of traditional W-band TWTs and offers a promising solution for high-capacity wireless backhaul, radar sensing, and other next-generation MMW applications requiring compact, efficient, and manufacturable power sources.

## 2. High-Frequency Characteristics

### 2.1. Structure of SSPP-Based SWS

**Figure 1:**
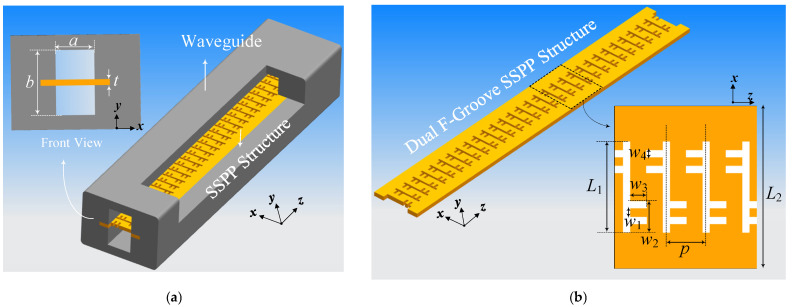
Structure of SSPP-based SWS: (**a**) View of the proposed SWS with cut shell; (**b**) Dual F-groove SSPP structure.

The proposed SWS adopts an SSPP configuration to achieve enhanced wave-beam interaction in low-voltage W-band TWT. The geometric layout of the SSPP-based SWS is illustrated in Figure 1. As shown in Figure 1a, the SSPP structure is embedded within a rectangular waveguide, with the upper metallic wall partially removed to expose the underlying planar slow-wave guiding structure for illustration. The cross-sectional view highlights the key dimensional parameters of the enclosing waveguide, with *a*, *b*, and *t* denoting the waveguide width, height, and the thickness of the SSPP structure, respectively. The core of the SWS is formed by a patterned planar metal structure featuring a periodic dual F-groove configuration, as depicted in Figure 1b. This SSPP structure supports tightly confined surface waves with significantly reduced phase velocity, which is essential for synchronization with low-energy electron beams. Each unit cell comprises a pair of asymmetric F-shaped slots etched on both sides of a central metallic strip, repeated periodically along the longitudinal z-direction with period *p*. Each F-groove is defined by geometrical parameters *L*_1_ and *L*_2_, representing the lengths of the vertical and horizontal branches, respectively. The widths of the grooves are labeled as *w*_1_, *w*_2_, *w*_3_, and *w*_4_, which jointly control the cut-off frequency and modal confinement of the SSPP wave. The dual F-groove design not only suppresses higher-order modes but also provides tunable dispersion through parametric adjustment, while remaining compatible with microfabrication tolerances. By integrating subwavelength periodic loading within a compact footprint, the proposed SSPP-based SWS enables efficient slow-wave propagation with strong field confinement. Furthermore, its open and symmetric design not only contributes to substantial phase velocity reduction and improved beam-wave coupling efficiency, but also inherently forms dual beam tunnels along the SSPP surface. These features make the structure particularly well-suited for miniaturized, low-voltage W-band TWTs, offering enhanced performance within a simple physical configuration.

### 2.2. The Dispersion Characteristics

**Figure 2:**
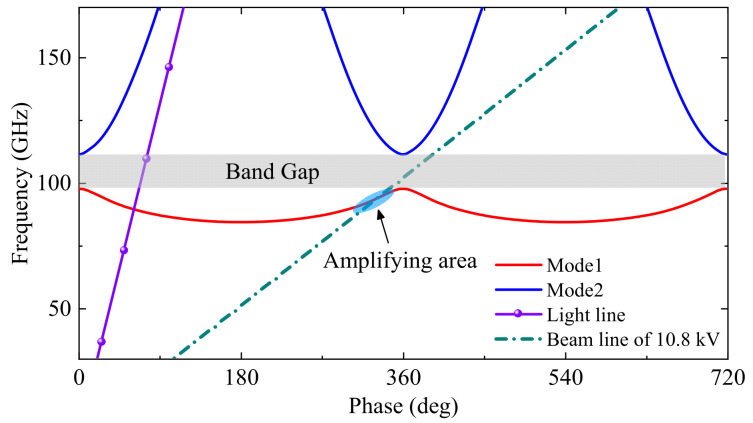
The ω−β diagram of the proposed SSPP-based SWS.

The dispersion characteristics of the proposed SSPP-based SWS are analyzed using eigenmode simulations with periodic boundary conditions, and its geometrical parameters are listed in Table 1. The calculated ω-β diagram of the unit cell is shown in Figure 2. Two distinct modes, identified as Mode 1 (fundamental) and Mode 2, are observed. Focusing on Mode 1, it exhibits a distinct transition between backward-wave and forward-wave propagation regimes. Specifically, in the 0°–180° phase-shift range, the slope of the dispersion curve is negative (∂ω/∂β < 0), indicating backward-wave behavior, whereas in the range of 180°–360°, the slope becomes positive (∂ω/∂β > 0), corresponding to a forward-wave mode. The beam line corresponding to a 10.8 kV beam is also plotted in Figure 2. The intersection of this beam line with the forward-wave region of Mode 1 defines the amplifying region, approximately within the 90–100 GHz band. Moreover, the bandgap between Mode 1 and Mode 2 helps mitigate undesired mode competition and suppress higher-order mode propagation, thereby improving spectral purity and device stability.

The normalized phase velocity *V_pc_* and the interaction impedance *K_c_* are fundamental metrics for evaluating the beam-wave interaction characteristics of an SWS. Specifically, *V_pc_* determines the required beam voltage for synchronism, whereas *K_c_* characterizes the efficiency of energy transfer between the RF wave and the electron beam. For synchronization in a TWT, the phase velocity of the SWS must match the velocity of the electron beam, which is determined by the beam voltage *U*_0_. This synchronism condition is described by the following relation:

(1) $$V_{pc}\approx \frac{v_{e}}{c_{0}}=\frac{\omega }{\beta c_{0}}=\sqrt{1-(\frac{1}{1+\eta U_{0}/c_{0}})}$$

where *η* is the electron charge-to-mass ratio, *β* is the phase constant, and *c*_0_ is the speed of light. Thus, a lower *V_pc_* implies a lower operating voltage.

The interaction impedance *K_c_* reflects the strength of coupling between the axial RF electric field and the beam. It is defined by [18]:

(2) $$K_{c}=\frac{{\left|E_{zn}\right|}^{2}}{2P{\beta_{n}}^{2}}$$

where *E_zn_* is the longitudinal electric field sampled along the beam path, *P* is the power flow, and *β_n_* is the phase constant of the *n*th spatial harmonic.

**Figure 3:**
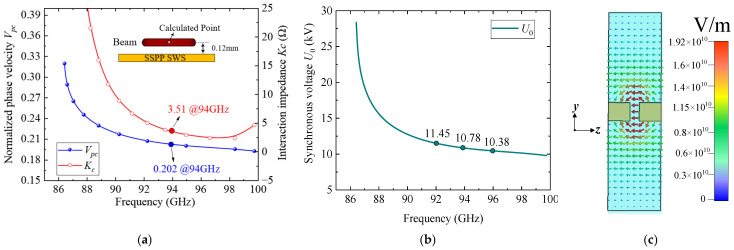
Eigenmode calculation results: (**a**) Normalized phase velocity and interaction impedance curves; (**b**) synchronous voltage curves; (**c**) Longitudinal electric field *E_z_* at *x* = 0 plane.

The normalized phase velocity *V_pc_* and interaction impedance *K_c_* of the proposed SSPP-based SWS are plotted in Figure 3a. The results show that *V_pc_* decreases monotonically from approximately 0.208 to 0.198 across the 92–97 GHz band, while *K_c_* consistently exceeds 2.65 Ω. The synchronous voltage *U*_0_, calculated from (1), is plotted in Figure 3b. As frequency increases, *U*_0_ decreases accordingly, reaching 10.8 kV at 94 GHz. This confirms the capability of the structure to achieve low-voltage beam-wave synchronization. The longitudinal electric field distribution at the cross-plane *x* = 0 is illustrated in Figure 3c, confirming the strong axial field confinement and beam-wave coupling efficiency within the SWS region.

**Figure 4:**
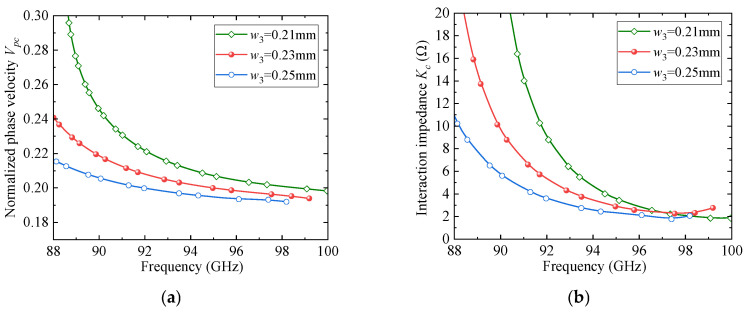
The effect of *w*_3_ on dispersion and impedance: (**a**) *V_pc_*; (**b**) *K_c_*.

**Figure 5:**
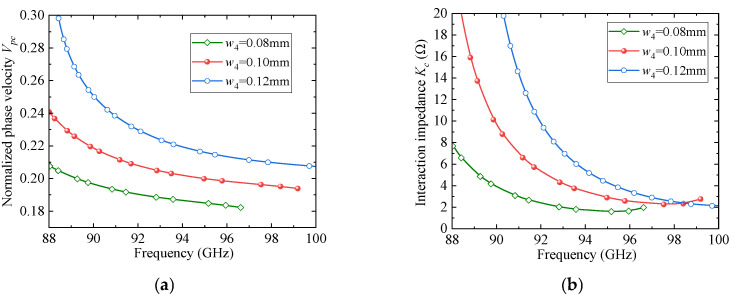
The effect of *w*_4_ on dispersion and impedance: (**a**) *V_pc_*; (**b**) *K_c_*.

**Figure 6:**
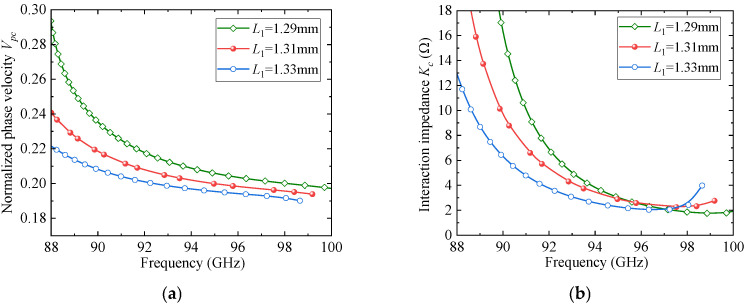
The effect of *L*_1_ on dispersion and impedance: (**a**) *V_pc_*; (**b**) *K_c_*.

To further clarify the rationale behind the parameter selection, a parametric sweep analysis was conducted for several key geometrical parameters of the proposed SSPP unit cell. Figure 4, Figure 5 and Figure 6 present the effects of the slot width and depth (*w*_4_ and *w*_3_) of the F-slot and the vertical slot length (*L*_1_) on both the normalized phase velocity (*V_pc_*) and the interaction impedance (*K_c_*). As shown in Figure 4, increasing *w*_3_ decreases the normalized phase velocity (*V_pc_*) but simultaneously reduces the interaction impedance (*K_c_*), indicating that excessively deep slots weaken field confinement. Similarly, Figure 5 and Figure 6 demonstrate that both *w*_4_ and *L*_1_ significantly affect dispersion and interaction impedance, with optimal intermediate values required to ensure strong beam-wave coupling while avoiding excessively high synchronism voltages. Among these parameters, *w*_4_ exhibits a more pronounced influence on dispersion characteristics. Based on these observations, the final parameter set was determined as an optimized compromise that ensures effective beam-wave interaction under low-voltage operation.

### 2.3. Transmission Characteristics Analysis

**Figure 7:**
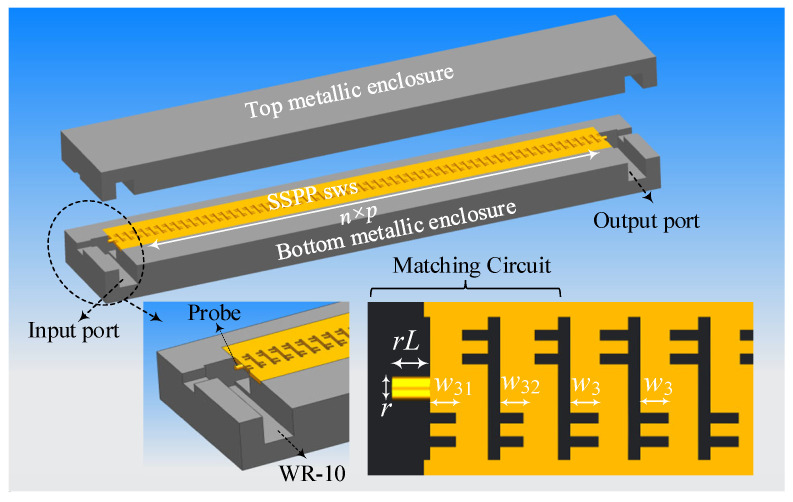
Assembly model of the proposed SSPP-based SWS with coupling structures.

To evaluate the transmission behavior of the proposed SSPP-based SWS, a full-wave model with 60-period units is established, as illustrated in Figure 7. The structure is enclosed within a metallic enclosure and excited through WR-10 standard waveguide ports. A circular probe was employed to excite the fundamental TE_10_ mode, ensuring mode purity and efficient coupling into the SSPP region. A dedicated matching circuit is integrated at the input and output interfaces to facilitate impedance transformation and minimize reflection. This matching section employed graded slot widths—from *w*_31_, *w*_32_ to the standard slot width *w*_3_—to ensure smooth modal evolution from the feeding waveguide to the periodic SSPP mode. The dimensional parameters, optimized to ensure efficient excitation and transmission, are listed as follows: *r* = 0.23 mm (probe diameter); *rL* = 0.31 mm (probe length); *w*_31_ = 0.21 mm; *w*_32_ = 0.21 mm. To account for fabrication non-uniformities, surface roughness, and unavoidable tolerances, a conductivity value of 3.0 × 10^7^ S/m was adopted in the simulation.

**Figure 8:**
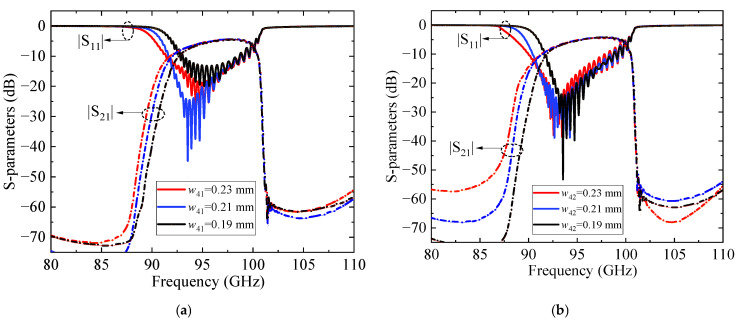
S-parameters for different: (**a**) *w*_41_; (**b**) *w*_42_.

**Figure 9:**
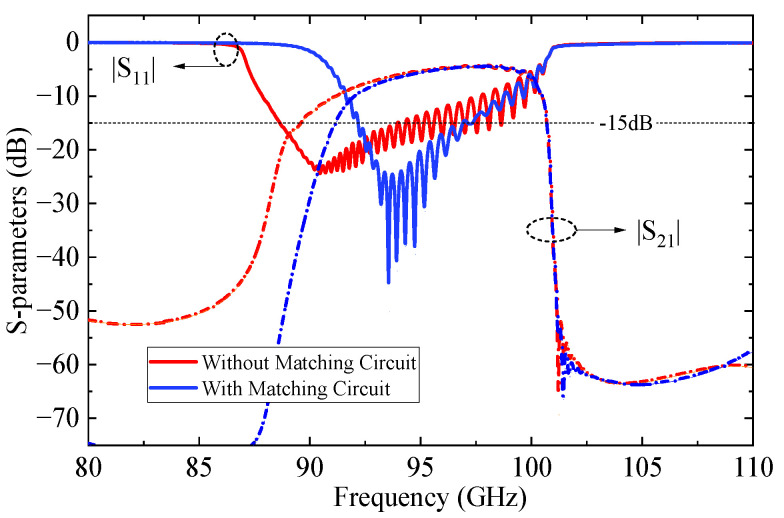
Impact of matching circuits on transmission characteristics.

Furthermore, S-parameter simulations were conducted using CST Microwave Studio 2023 (CST MWS) [27] to characterize the transmission and reflection responses of the structure. The influence of the matching section was systematically studied by varying the intermediate slot widths, as shown in Figure 8. The results indicate that, for both *w*_41_ and *w*_42_, the in-band impedance matching initially improves and then degrades as the width increases, accompanied by a reduction in bandwidth. Figure 9 compares the transmission characteristics with and without the matching circuit. The comparison results indicate that the introduction of the matching circuit significantly improves the return loss (RL) in the 91–97 GHz frequency band, achieving a reflection coefficient better than −15 dB and a minimum insertion loss (IL) of 0.8 dB, thereby demonstrating excellent transmission performance.

**Figure 10:**
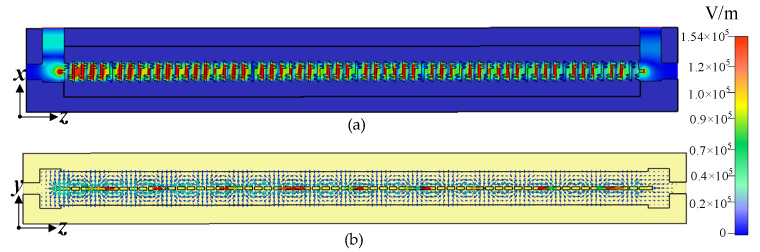
Electric field distribution at 94 GHz of the transmission model: (**a**) at *y* = 0; (**b**) at *x* = 0.

Figure 10 presents the electric field distributions at 94 GHz in transverse and longitudinal cross-sections (i.e., *y* = 0 and *x* = 0 planes). The results clearly show strong axial field localization within the SSPP groove regions (Figure 10a), and periodic slow-wave behavior along the beam path (Figure 10b). These features confirm the structure’s suitability for high-efficiency beam-wave interaction with enhanced mode purity and low loss.

## 3. Analysis of Beam-Wave Interaction

**Figure 11:**
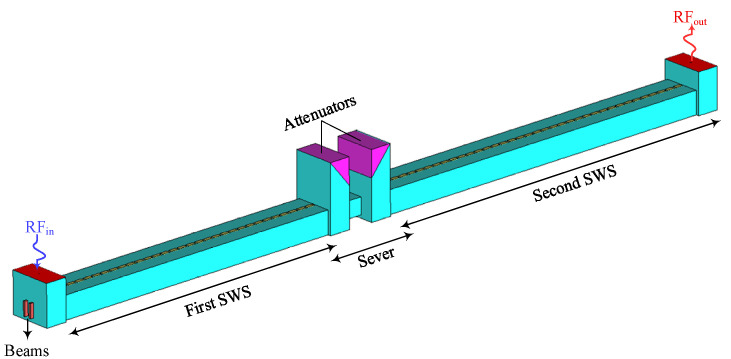
Beam-wave interaction model.

**Figure 12:**
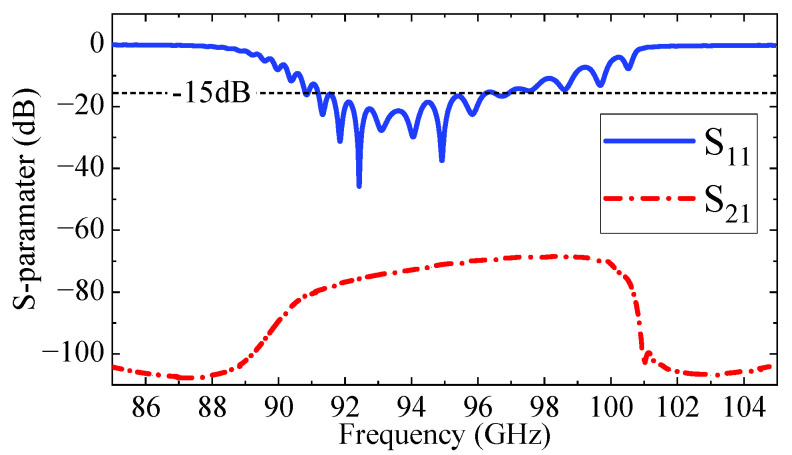
Transmission characteristics of the beam-wave interaction circuit.

The beam–wave interaction characteristics of the proposed W-band TWT were investigated through three-dimensional particle-in-cell (PIC) simulations in CST Particle Studio 2023 (CST PS). In practical TWT designs, the slow-wave circuit is typically divided into two sections, with a sever inserted to suppress backwaves and parasitic oscillations. Usually, the sever is placed in the rising portion of the small-signal gain curve, while maintaining sufficient margin from the saturation region, and is thus located near the midpoint of the circuit or slightly upstream [28,29,30]. After extensive simulations and optimizations, the complete circuit, as illustrated in Figure 11, consists of two sections with 31-and 37-unit cells, respectively, separated by a sever to suppress the potential oscillations. A pair of BeO-based attenuators (with a relative permittivity of 6.5 and a loss tangent of 0.8) is introduced at the sever location to enhance stability. The total circuit length, including the input and output coupling transitions of the structure, is 45.34 mm. Figure 12 shows the transmission characteristics of the beam-wave interaction circuit. The reflection coefficient (*S*_11_) remains below −15 dB within the 91–100 GHz range, indicating good impedance matching across the operational bandwidth. Meanwhile, the transmission coefficient (*S*_21_) remains below −68.7 dB across the entire frequency band, demonstrating excellent cutoff attenuation. This performance effectively suppresses undesired signal reflections and mitigates the risk of self-oscillation within the structure.

In the simulation, a 2 × 250 mA dual-sheet beam operating at 11.9 kV was considered. The beam comprises two independent beam bundles, each with a cross-sectional area of 0.12 mm × 0.8 mm. The axis of each bundle is positioned 0.05 mm from the adjacent metal plate. The resulting average current density is 260.4 A/cm^2^, which is a relatively moderate value for a sub-terahertz TWT. Meanwhile, a longitudinal magnetic field of 1.0 T was applied to ensure beam focusing and suppress divergence. Similar magnetic field strengths have also been adopted in previously reported MMW TWTs, confirming the feasibility of this assumption [31,32,33]. The optimization of compact high-field focusing structures will be considered in future work. The PIC simulations were carried out on a workstation equipped with an Intel Core i7-10700K CPU, 32 GB RAM, and an NVIDIA GeForce RTX 3070 GPU, with GPU acceleration used to improve efficiency and shorten simulation time.

**Figure 13:**
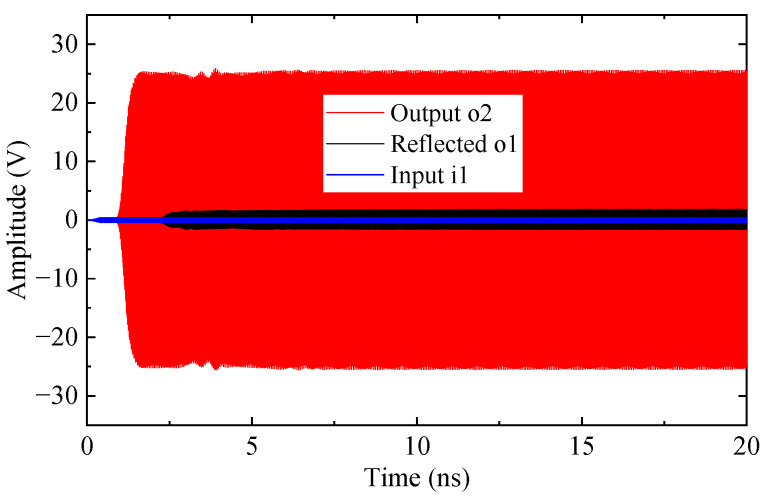
Time-domain signals of the beam-wave interaction at 94 GHz.

Figure 13 presents the time-domain signals of the TWT at 94 GHz utilizing the SSPP-based SWS, including the input at port 1 (green), reflection at port 1 (black), and output at port 2 (red) responses. A steady state output is established after 2 ns, and no oscillation is detected throughout the 20 ns simulation interval. The output power reaches 340.6 W with an input power of 0.09 W, yielding a gain of 26.1 dB and an electronic efficiency of 5.48%.

**Figure 14:**
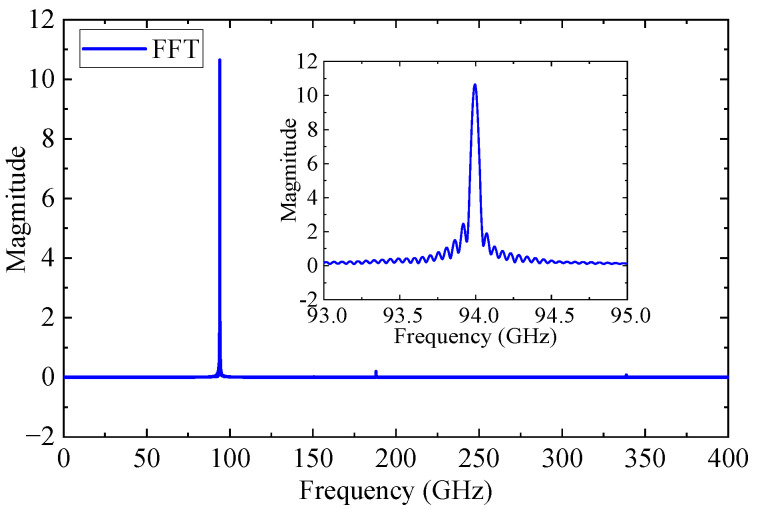
Frequency spectrum of the output signal.

To verify stable amplification, a Fourier transform was applied to analyze the output spectrum. As shown in Figure 14, the spectrum exhibits a dominant peak at 94 GHz with only a weak second harmonic at 188 GHz, indicating a minimal risk of oscillation.

**Figure 15:**
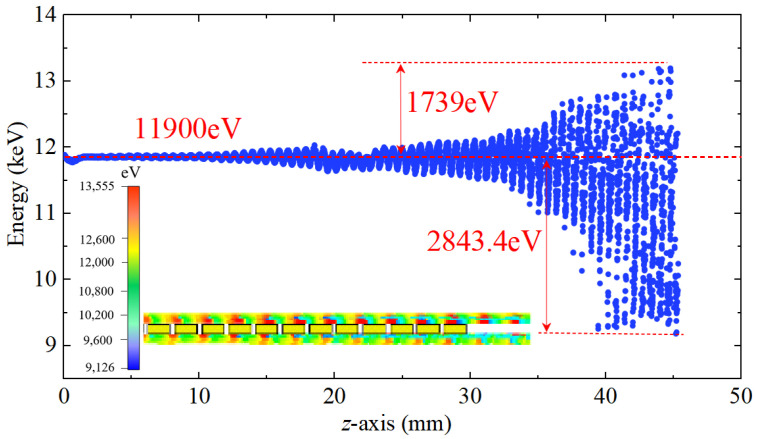
Phase space plot of the bunched electron beam at 15 ns.

Figure 15 presents the phase-space energy distribution of electrons along the *z*-direction at 15 ns, when the modulation reaches steady state. The beam is initially centered at 11.9 keV, and as it propagates through the interaction region, progressive energy modulation is observed. The beam undergoes substantial energy exchange, exhibiting maximum deviations of approximately +1739 eV and −2843.4 eV from the initial energy. This pronounced modulation demonstrates efficient beam-wave interaction within the SWS.

**Figure 16:**
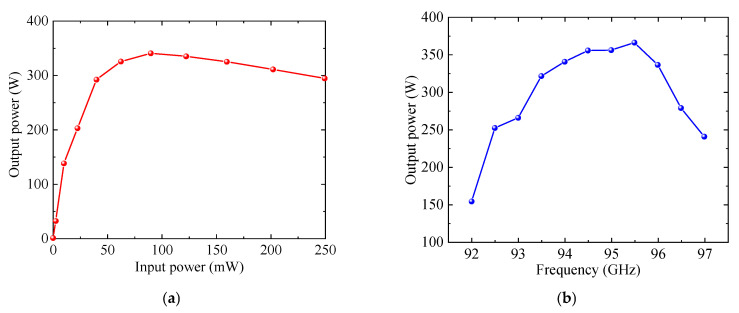

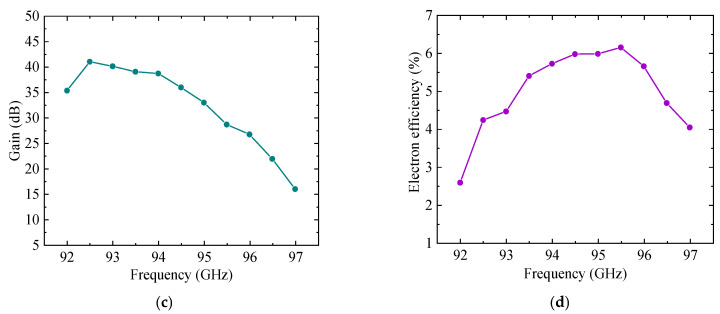
Beam-wave interaction performance: (**a**) Output power versus input power at 94 GHz; (**b**) Saturated output power versus frequency; (**c**) Gain versus frequency; (**d**) Electron efficiency versus frequency.

Comprehensive PIC simulations were performed to investigate the beam-wave interaction characteristics of the proposed W-band TWT, with the results depicted in Figure 16. Figure 16a illustrates the output power versus input power from Port 1 to Port 2 at 94 GHz. As the input power increases from 0 to 250 mW, the output initially exhibits linear growth and subsequently enters saturation. Peak saturated output power is achieved at an input power of approximately 90 mW, yielding 340.6 W at port 2. Figure 16b presents the saturated output power versus frequency across the 92–97 GHz frequency range. At around 95.5 GHz, the output power reaches a maximum value of 366.1 W, indicating effective beam-wave synchronization and efficient interaction at this frequency point. As shown in Figure 16c, the gain versus frequency curve exhibits a peak gain of over 40 dB around 92.5 GHz, after which the gain gradually decreases. Figure 16d depicts the electron efficiency versus frequency. A maximum efficiency of 6.15% is achieved at 95.5 GHz. Table 2 presents a performance comparison between the current SSPP-based design and representative W-band TWTs utilizing conventional SWSs. The results show that our design achieves comparable or superior performance while requiring a significantly lower operating voltage. These advantages clearly validate that the designed TWT achieves high power and efficiency performance under low-voltage operation, making it a promising solution for W-band amplification.

## 4. Conclusions

A W-band low-voltage TWT based on a dual-sheet beam and an SSPP-based SWS was developed and analyzed. The SSPP-based SWS offers strong axial field confinement, high interaction impedance, and reduced synchronization voltage, enabling efficient operation below 12.0 kV. The dual-sheet beam configuration enhances output power without increasing device cross-sectional area, making it suitable for compact and lightweight systems. Beam-wave interaction simulations show that, within the 92–97 GHz band, the output power exceeds 340 W, the gain is above 26 dB, and the efficiency reaches 6.15%, with stable operation and no self-oscillation. These results demonstrate that the proposed TWT provides a compact, manufacturable, and high-performance solution for W-band power amplification in next-generation millimeter-wave communication systems.

## Figures and Tables

**Table 1:** Dimension parameters for SSPP-based SWS.

Parameters	Dimension (mm)	Parameters	Dimension (mm)
*L*_1_	1.31	*L*_2_	2.80
*p*	0.57	*w*_1_	0.12
*w*_2_	0.44	*w_3_*	0.23
*w*_4_	0.10	*a*	1.31
*b*	2.15	*t*	0.20

**Table 2:** Performance comparison of this work with traditional SWS.

Reference	Type	f (GHz)	U (kV)	Pout (W)	Gani (dB)	Efficiency
[8]	FWG	91–98	21.5	>600 ^#^	>29 ^#^	>6.7% ^#^
[9]	FWG	90–94.5	21.9	>450 ^#^	>29.3 ^#^	>7.9% ^#^
[12]	SGW	90–95	22.6	>450 *	>26.5 *	>4.5% *
[13]	FWG	90–100	23.4	>1000	>20.1 *	>7.18% *
[16]	FWG	92–98	21.75	>200 ^#^	>32.5 ^#^	>5.5% ^#^
[17]	SWG	92–97	20.6	>200 *	>30.2 *	>5.4% *
[19]	MTM	91.5–95.5	8.50	<50	>30.0	<3.92%
This work	SSPP	93–96	11.9	>265	>26.7	>4.46%

*: Estimated from the papers, ^#^: Hot-test results

## Data Availability

No new data were created or analyzed in this study. Data sharing is not applicable to this article.
